# Surgical Treatment of Paediatric Thalamic Gliomas—Single-Centre Experience

**DOI:** 10.3390/brainsci14020141

**Published:** 2024-01-29

**Authors:** David Krahulik, Filip Blazek, Matej Halaj, Lumir Hrabalek, Eva Stepanova, Zdenek Pavelka, Marie Rohanova

**Affiliations:** 1 Department of Neurosurgery, University Hospital Olomouc, 77900 Olomouc, Czech Republic; filip.blazek@fnol.cz (F.B.); matej.halaj@fnol.cz (M.H.); lumir.hrabalek@fnol.cz (L.H.); 2 Department of Paediatric Neurology, University Hospital Ostrava, 70852 Ostrava, Czech Republic; eva.stepanova@fno.cz; 3 Department of Paediatric Oncology, University Hospital Brno, 66263 Brno, Czech Republic; pavelka.zdenek@fnbrno.cz; 4 Department of Paediatrics, University Hospital Olomouc, 77900 Olomouc, Czech Republic; marie.rohanova@fnol.cz

**Keywords:** paediatric thalamic gliomas, bithalamic gliomas, radical surgery of gliomas in children

## Abstract

The surgical treatment of paediatric thalamic gliomas has been burdened with high morbidity, and these lesions were often considered inoperable. With new approaches and intraoperative technologies, we can remove tumours once deemed inoperable. In our single centre, we have operated on 11 paediatric patients over the course of 8 years. We have performed eight GTR resections and three intended subtotal resections. The postoperative neurological deficit ranged from mild to very severe for motor weakness and none to severe for aphasia after surgery, with all of the patients improving at 3-month follow-up. Radicality in the surgical approach to thalamic gliomas in children has shown significant benefits when compared to more conservative approaches. For children with LGGs, extensive surgical resection is associated with improved prognosis and longer progression-free survival. However, it does not yield the same proportional benefit for HGGs due to its aggressive nature and worse outlook.

## 1. Introduction

Paediatric neoplasms originating in the thalamic region are a subset of brain tumours affecting one of the critical neural structures located deep within the cerebral hemispheres [1]. Children diagnosed with thalamic gliomas face unique challenges due to the tumour’s location and the sensitive period of neural development. Despite being relatively rare, accounting for less than 5% of all intracranial tumours in children, these tumours pose significant clinical management dilemmas, given their location, potential symptoms, and complex treatment decisions required [2].

They are more commonly located unilaterally, while bithalamic gliomas represent less than 1% of all brain tumours. Most paediatric thalamic gliomas arise directly from the thalamus, while a smaller number grow from the junction of the cerebral peduncle and thalamus; these are called thalamopeduncular tumours.

In the past, diagnosing thalamic gliomas in children led to a more conservative approach due to the proximity of vital neuroanatomical structures such as the cortico-spinal tract, internal capsule, and neurovascular structures [3]. The risks of the surgery outweighed the potential benefits. At the same time, it remains true that damage to the thalamus or surrounding structures can cause severe disability, with the eventual improvement and development of surgical techniques and accompanying technologies lessening this risk [4,5].

More detailed preoperative imaging allows for more accurate and careful planning, while perioperative online image guidance and intraoperative neuromonitoring allow for more diligent yet more extensive resection associated with improving surgical mortality and morbidity rates [4].

While even with these advances, the overall prognosis remains poor for some cases, children with low-grade gliomas (LGGs) are very likely to reach adulthood [6]. In the case of histologically confirmed pilocytic astrocytoma with total surgical resection, these patients can even be completely cured without any adjuvant chemotherapy or radiotherapy. With this in mind, the surgery should provide the maximal possible resection. At the same time, with the guidance of intraoperative neuromonitoring and online image guidance, it should maintain or improve the preoperative neurological status [7].

This study presents our data collected since 2015 in a single neurosurgical centre in the Czech Republic, focusing on the radicality of the surgery while achieving the best possible clinical outcome.

## 2. Materials and Methods

In this single-centre prospective study, 11 patients in total were included in the period of 2015–2023. In this period, all presented cases underwent surgery and were thus included in this study.

The clinical and imaging attributes of the patients were examined, encompassing their clinical symptoms, prior treatments, immediate post-surgery outcomes, and eventual outcomes. Every patient underwent a contrast-enhanced MRI evaluation. We also incorporated diffusion tensor echo planar imagery with tractography [7], which was analysed preoperatively. Two independent neuroradiologists assessed all imaging studies—both before and after surgery. These assessments focused on tumour volume (measured in cm^3^), tumour location and spread, imaging characteristics, displacement or infiltration of the corticospinal tract (CST) on T2-weighted and FLAIR MR images, and signs of hydrocephalus [8]. Distinctive imaging features such as hydrocephalus, lesion heterogeneity and cystic components, calcifications, swelling, and tissue death were identified on T1- and T2-weighted MRI scans.

Immediate post-surgery MRI scans, with postcontrast, were taken within 24 h for all patients. Based on the imaging results, the degree of tumour removal was categorised as STR (more than 90% resected) or GTR (complete removal with no remaining tumour) [9,10]. These images were also used to identify any post-surgical complications.

Follow-up MRI scans performed at the three-month mark post-surgery, then biannually for three years, and annually for the next three years were reviewed to monitor for any recurrence or enlargement of any leftover tumour post-surgery. Naturally, the MRI follow-up schedule was adjusted based on the patient’s clinical status changes.

### 2.1. Surgical Approach

Every patient underwent surgery aimed at achieving complete removal of the tumour through microsurgical techniques. The surgical procedure involved the systematic utilization of neuronavigation and intraoperative monitoring of motor and somatosensory evoked potentials. The choice of the surgical approach was determined by the thalamic lesion’s location and its impact on surrounding structures. The approach was carefully planned to navigate through nonessential brain tissue, ensuring avoidance of the posterior limb of the internal capsule or the CST while prioritizing the maximal preservation of displaced or compressed typical thalamic structures. Depending on the tumour localisation, we usually chose the transcortical approach, with the interhemispheric approach used sporadically. Where applicable, we used the 5-aminolevulinic acid hydrochloride fluoroscopy perioperatively to judge the extent of resection. For low-grade tumours, where 5-ALA is not used, we based the extent of resection on macroscopic tissue difference and using the contrast enhanced preoperative MRI.

### 2.2. Follow-Up and Adjuvant Therapy

Based on histological findings, clinical evaluation, and postoperative imaging, a multidisciplinary team comprising neurosurgeons, paediatric oncologists, and radiotherapists determined the optimal treatment. For low-grade tumours, the standard approach involved MRI follow-up at three months without additional treatment. High-grade tumours, identified through histology, were treated with radiotherapy and various chemotherapy protocols [8,11]. Tumour recurrence was defined as the emergence of previously absent contrast-enhancing tissue in the surgical cavity or an increase in the volume of a known postoperative tumour residue [12].

## 3. Results

**Figure 1:**
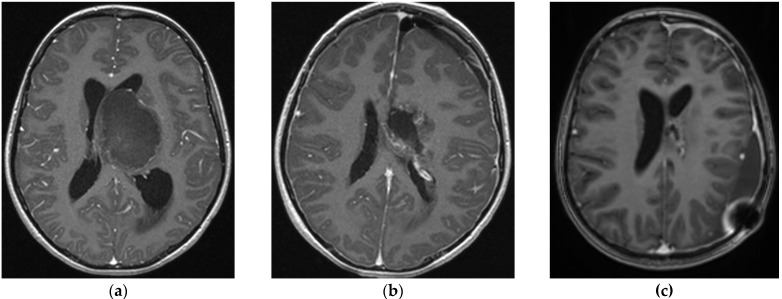
Eleven-year-old girl with acute clinical worsening due to an intratumoral haemorrhage. (**a**) Pre-op imaging; (**b**) GTR; (**c**) 6 m follow-up with no recurrence and VP drainage in place due to hydrocephalus.

Since 2015, 11 children with thalamic tumours have undergone an operation in our centre. There were six girls and five boys. The mean age at the time of tumour resection was 12.3 years (range 4–18 years). One of these children was referred after a previous surgical biopsy. The mean follow-up was 46 months for low-grade tumours and 14 months for high-grade tumours. Two (18%) patients died during follow-up, seven (64%) are alive in complete remission, and two (18%) are alive with stable disease (Table 1). Seven tumours were right-sided, and four were left-sided. All patients were symptomatic at the time of treatment. Motor deficits were the most common form of presentation (11 cases), with symptoms of increased intracranial pressure (ICP) (2 cases—18%) related to hydrocephalus (1 case—9%) or to the volume of the tumour itself (1 case—9%). One patient (9%) was admitted under emergency status with a Glasgow Coma Scale (GCS) score of 5 due to an intratumoral haemorrhage and acute hydrocephalus (Figure 1). Less frequent symptoms were sensory deficit (two patients—18%), epilepsy (one patient—9%), and aphasia (one patient—9%). The detailed histology is presented in Table 2.

### 3.1. Complications

Postoperative complications, adverse events, and clinical status six months after surgery were classified as transient or permanent and are summarised in Table 3.

### 3.2. Outcome in Patients with Low-Grade Tumours

**Figure 2:**
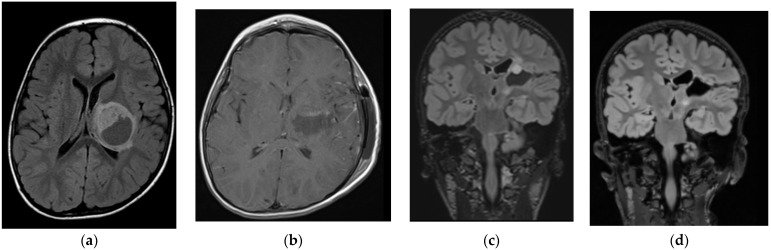
Four-year-old boy with pilocytic astrocytoma. (**a**) Pre-op imaging; (**b**) GTR; (**c**) small recurrence after 2 years; (**d**) second GTR.

Low-grade tumours accounted for 72.7% of lesions in our series (Table 1). A GTR was achieved in six cases and STR in two cases. The mean follow-up was 46 months. STR resected tumours are controlled every three months, and there is no growth of remnant tumours so far. The tumour recurred in one patient, and GTR was achieved via the same surgical approach as the first operation (Figure 2).

### 3.3. Outcome in Patients with High-Grade Tumours

Three patients presented with high-grade tumours were in our series (Table 1). The mean follow-up was 14 months. GTR was achieved in two patients and STR in one patient. All patients had chemotherapy and radiotherapy. One patient had also added therapy with vinorelbine and nimotuzumab but died because of the disease progression 15 months after the initial surgery.

The second patient had chemotherapy and radiotherapy and died because of the disease progression 21 months after the initial surgery. The last patient is now six months after initial surgery with no evidence of disease. She had radiotherapy, chemotherapy, and experimental biological treatment.

## 4. Discussion

The management of paediatric thalamic gliomas is both intricate and multidisciplinary. The deep-seated location of the thalamus, coupled with its integral role in relaying sensory and motor signals and regulating consciousness, sleep, and alertness, makes surgical intervention challenging. The decision to proceed with a surgical approach is based on several factors, including the tumour’s size, location, pathology, and clinical symptoms [1,3,13].

Historically, a fully conservative approach was usually chosen, with stereotactic biopsy once deemed the only safe surgical procedure for many thalamic gliomas [14]. While biopsy aids in obtaining tissue samples [15], determining the tumour’s grade and guiding the subsequent treatment, it does not provide a complete curative means.

With the advances in microsurgical and accompanying techniques, resection is now widely accepted as the primary and best line of treatment, providing the best possible results.

Advancements in neuronavigation systems utilising preoperative and intraoperative imaging enhance the surgeon’s precision when navigating the brain’s complex anatomy [16]. Another technique widely used, neurophysiological monitoring, provides the real-time monitoring of neurological functions, such as motor and sensory pathways, aiding in the avoidance of damaging these critical structures during tumour resection while allowing for maximal surgical radicality.

Overall, paediatric thalamic gliomas, especially high-grade variants, have a poorer prognosis than similar tumours in more accessible brain regions [17,18]. The deep location, coupled with the adjacent critical structures of the thalamus, often limits aggressive surgical resection. This decision-making process is further complicated by considerations specific to the paediatric population, such as the potential impact of treatment on the developing brain and its long-term cognitive and developmental outcomes.

The nature of paediatric low-grade gliomas, especially pilocytic astrocytoma, is favourable, with excellent survival prognosis compared to adults and HGGs in children [2,11]. Overall survivability rates at ten years are around 85% [19]. The main difference from their adult counterparts is that juvenile LGGs seldom undergo malignant transformation, even in adulthood. Overall, the long-term mortality for pilocytic astrocytoma histology is around 2%. Thus, paediatric patients with LGGs are highly likely to reach advanced adulthood without the added morbidity of their glioma. All of these facts must be considered when creating a treatment plan, aiming at maximal tumour resection and control while minimising the related treatment complications.

The main line of treating paediatric thalamic gliomas is surgery. Providing a comprehensive approach allows for obtaining histological samples while removing the maximal possible mass of the tumour, relieving its mass effect, and, if total resection is possible, further increasing the overall chance of survival. The primary aim of the surgery is maximal safe resection (ideally GTR on postoperative MRI) while preserving the known and perioperatively monitored vital structures. However, it is essential to note that thalamic tumours present technical challenges due to their deep location and relativity to these structures. The chosen surgical approach for all patients included in this study was dictated by the tumour position in relation to the local anatomy. The interhemispheric, trans-callosal route is the most commonly used approach in targeting these tumours [3,6,7]. This surgical approach provides ideal exposure and microsurgical access for more medially located tumour masses. For lesions located more laterally, a transtemporal approach was used. Combined, two staged procedures are needed for large lesions, such as those extending to the posterior fossa.

Other treatment options are sometimes considered, such as chemotherapy for unresectable tumours or in cases of partial resection. Drugs like vincristine and carboplatin are commonly used, especially in younger children where radiation concerns are more pronounced. Radiation therapy, while effective, is often deferred in the youngest patients due to concerns about cognitive and developmental side effects. Focal radiation is considered for older children or as a last line of treatment when the tumour is progressive despite all previous attempts.

In the future, different emerging techniques might lead to even more targeted therapies and reduce the invasiveness of the existing techniques. Molecular profiling of the tumours, enabling us to understand the genetic and molecular characteristics of the gliomas, might offer better insights into the prognosis and guide more targeted therapies [20]. The use of robot-assisted surgery, though at this moment in nascent stages for neurosurgery, might offer even greater precision and reduce invasiveness.

Our data provided here indicate an imperative role of aggressive surgical resection in most cases. Complete tumour resection provides a cure in cases of LGGs, mainly pilocytic astrocytoma, and when performed following the newest findings and techniques, does not significantly increase the surgical morbidity nor long-term neurological disability [21].

## 5. Conclusions

The neurosurgical treatment of paediatric thalamic gliomas is a complex endeavour that requires meticulous planning, advanced techniques, and a multidisciplinary approach. While surgery offers potential for cure or control, the risks associated with intervention in such a critical brain region necessitate a careful balance between achieving maximal tumour removal and preserving neurological function. As indicated in our study group, complete resection often leads to the best long-term outcomes when feasible.

## Figures and Tables

**Table 1:** Cohort of patients included in our study.

Case #	Age y/o	Histology	Grade of Resection	Adjuvant Treatment	Complications	Follow Up in Months	Recurrence	Clinical Status
**Low Grade**								
1	9	Pilocytic astrocytoma	GTR	No	No	96	No	ANED
2	17	Pilocytic astrocytoma	GTR	No	No	84	No	ANED
3	7	Pilocytic astrocytoma	GTR	No	No	12	No	ANED
4	15	DNET	GTR	No	No	60	No	ANED
5	4.6	Pilocytic astrocytoma	GTR 2×	No	No	40	Yes	ANED
6	16	Pleomorphic xanthoastrocytoma	STR	No	No	42	No	ASD
7	11	Pilocytic astrocytoma	GTR	No	No	18	No	ANED
8	5	Ganglioglioma	STR	No	No	16	No	ASD
**High Grade**								
9	18	Astrocytoma WHO gr. 4	GTR	CR *	No	21	Yes	DOD
10	15	Mut. diffuse midline glioma	STR	CRI *	Hydrocephalus—VP drainage	15	Yes	DOD
11	11	Mut. diffuse midline glioma	GTR	CRI *	Hydrocephalus—VP drainage	6	No	ANED

* used abbreviations: #—number; CR—chemotherapy and radiotherapy; CRI—chemotherapy, radiotherapy, and immunotherapy; ANED—alive with no evidence of disease; DOD—died of disease; ASD—alive with signs of disease.

**Table 2:** Detailed histology.

Case #	Histology
**Low-Grade**	
1	Pilocytic astrocytoma IDH 1,2 negative, BRAF-KIAA fus. positive
2	Pilocytic astrocytoma IDH 1,2 negative, BRAF-KIAA fus. positive
3	Pilocytic astrocytoma IDH 1,2 negat, BRAFv600 mut
4	Dysembryoplastic neuroepithelial tumour
5	Pilocytic astrocytoma IDH 1,2 negat, BRAF-KIAA fus. pos.
6	Pleomorphic xanthoastrocytoma BRAFv600 mut, homozyg. deletion CDKN2A
7	Pilocytic astrocytoma IDH 1,2 negat, BRAFv600 mut
8	Ganglioglioma
**High-Grade**	
9	Astrocytoma WHO gr. 4 iDH wild type
10	Diffuse mutant midline glioma, gr. 4, mut.H3-3A K27M
11	Diffuse mutant midline glioma, gr. 4, mut.H3-3A K27M

used symbols #—number.

**Table 3:** Neurological deficit before and immediately after surgery and upon a 3-month follow-up.

Case #	Motor Weakness Before/After Surgery	Aphasia Before/After Surgery	Motor Weakness/3 mo	Aphasia/3 mo	Karnofsky/6 mo
1	+/++	−/−	+	−	90
2	+/+	−/−	−	−	100
3	+/+	−/−	−	−	100
4	+/++	−/+	+	−	90
5	+/+	−/−	+	−	90
6	+/+	−/−	−	−	100
7	+/++	−/+	−	−	100
8	+/+	−/−	−	−	100
9	+/++	−/−	+	−	90
10	+/+++	−/+	++	−	70
11	+/++	+/++	+	−	90

#—number; deficit evaluation: none, −; mild, +; severe, ++; very severe, +++.

## Data Availability

The datasets used and/or analysed during the current study are available from the corresponding author on reasonable request. The data are not publicly available due to the sensitive information contained.
